# ASO Author Reflections: Impact of Frailty on Liver Surgery

**DOI:** 10.1245/s10434-024-15662-6

**Published:** 2024-06-21

**Authors:** Sorinel Lunca, Stefan Morarasu

**Affiliations:** 1 2nd Department of Surgical Oncology, Regional Institute of Oncology (IRO), Iasi, Romania; 2 Department of Surgery, Grigore T Popa University of Medicine and Pharmacy Iasi, Iasi, Romania

## Past

The increasing life expectancy around the world exerts pressure on surgical services as more and more elderly patients are recommended major operations when alternative treatments are known to have poor results. In both primary cancer and liver metastasis, surgery is the mainstay of treatment, proven to impact survival rates; however, major liver resection is one of the most complex and high-risk abdominal interventions. For the above reasons, research is needed to better define and stratify frail patients and their outcomes after major liver surgery.^1–5^

## Present

This is the first meta-analysis to compare surgical outcomes between frail and nonfrail patients and underscores the importance of in-depth preoperative evaluation of patients undergoing major liver resections. Frail patients undergoing oncological liver resections have a higher rate of postoperative morbidity, including surgical, medical complications, and postoperative liver failure, with a longer hospital stay and more frequent readmissions. Frail patients have a significantly higher rate of 30-days mortality compared with nonfrail patients. Comparatively, frail patients are older and show a lower preoperative albumin level, suggestive of a poorer nutritional status.

## Future

Frailty is defined in many ways by various scores/tools. Before general adoption of frailty in the preoperative evaluation of liver surgery candidates, we must establish an unanimously accepted score for quantifying frailty. This will decrease interstudy heterogeneity, improve extraction of data, multicenter data comparison and, finally, will bring stronger research—a building block for routine implementation of frailty in the preoperative workup.
